# Healthcare Professionals Describe Difficulties Encountered When Breaking Bad News to Oncology Patients: An Italian Observational Study

**DOI:** 10.3390/nursrep16010004

**Published:** 2025-12-23

**Authors:** Stefano Botti, Luana Conte, Marco Cioce, Laura Orlando, Enrica Tamagnini, Chiara Cannici, Angela Capuano, Valentina De Cecco, Ludovica Panzanaro, Nicola Serra, Giorgio De Nunzio, Roberto Lupo, Elsa Vitale

**Affiliations:** 1 Health Professions Research Unit, Azienda USL-IRCCS of Reggio Emilia, Via Amendola 2, 42122 Reggio Emilia, Italy; enrica.tamagnini@ausl.re.it; 2 Hematology Unit, Azienda USL-IRCCS of Reggio Emilia, Viale Risorgimento 80, 42123 Reggio Emilia, Italy; 3 Department of Physics and Chemistry, University of Palermo, Viale delle Scienze, Ed. 17, 90128 Palermo, Italy; luana.conte@unipa.it; 4 Department UOC SITRA, Fondazione Policlinico Universitario A. Gemelli IRCCS, Largo Agostino Gemelli 8, 00136 Rome, Italy; marco.cioce@policlinicogemelli.it; 5 Department of Nursing, Oncology Institute of Southern Switzerland, Ente Ospedaliero Cantonale (EOC), Via A. Gallino 12, 6500 Bellinzona, Switzerland; laura.orlando@eoc.ch; 6 Division of Hematology, Azienda Ospedaliero-Universitaria SS. Antonio e Biagio e Cesare Arrigo di Alessandria, Via Venezia 16, 15121 Alessandria, Italy; chiara.cannici@ospedale.al.it; 7 Healthcare Directorate, AORN Santobono-Pausilipon, Via T. Ravaschieri 8, 80128 Naples, Italy; a.capuano@santobonopausilipon.it; 8 Department of Onco-Haematology and Cell and Gene Therapy, Bambino Gesù Children’s Hospital IRCCS, Piazza Sant’Onofrio 4, 00165 Rome, Italy; valentina.dececco@opbg.net; 9 C.R.A.P. Carrubo, Sol Levante S.r.l., Via Roma 156, 74020 Avetrana, Italy; ludovica.panzanaro@hotmail.it; 10 Department of Neuroscience, Reproductive Sciences and Dentistry-Audiology Section, University Federico II of Naples, Via Pansini 5, 80131 Naples, Italy; nicola.serra@unina.it; 11 Laboratory of Biomedical Physics and Environment, Department of Mathematics and Physics “E. De Giorgi”, University of Salento, Via per Arnesano, 73100 Lecce, Italy; giorgio.denunzio@unisalento.it; 12 Advanced Data Analysis in Medicine (ADAM), Laboratory of Interdisciplinary Research Applied to Medicine (DReAM), University of Salento and ASL (Local Health Authority), Piazza Filippo Bottazzi, 73100 Lecce, Italy; 13 San Giuseppe da Copertino Hospital, ASL (Local Health Authority), Via Carmiano, 73043 Lecce, Italy; roberto.lupo@asl.lecce.it; 14 Directorate of Health Professions and Nursing, ASL Bari, Via Armando Diaz 136, 70123 Bari, Italy; elsa.vitale@asl.bari.it

**Keywords:** bad news, communication, stress, nursing, healthcare professionals, oncology, hematology

## Abstract

**Background:** Many nurses and physicians report difficulties with breaking bad news to their patients due to the lack of adequate skills and training. This study aimed to explore the communication skills, knowledge, and self-perceived difficulties of healthcare professionals working in oncology and hematology settings in Italy, in relation to their self-perceived stress levels when communicating bad news. **Methods:** An “ad hoc” questionnaire and the Perceived Stress Scale were administered online to both physicians and nurses registered by two important professional associations between October 2023 and September 2024. **Results:** A total of 221 Italian physicians and nurses were enrolled in the study. Most participants reported learning how to conduct difficult conversations from a mentor (61.1%) or through specific courses (56.6%). However, many of the recruited subjects declared having difficulty in giving bad news to the patient and family members (84.2%), and many of them did not know the SPIKES method (63.8%). A moderate level of stress was perceived by the great majority of participants, and the stress level was significantly increased in healthcare professionals who had difficulties in using evidence-based tools (e.g., SPIKES) for bad news communication. Moderate stress was “often” experienced by participants when presenting themselves during the first approach (*p* = 0.006), when attempting to anticipate the patient’s reactions (*p* = 0.044), when the patient refused to receive information (*p* = 0.006), when they had to remain assertive and confident regardless of the patient’s response (*p* = 0.013), and when managing post-communication consequences (*p* = 0.012). **Conclusion:** The limited knowledge and application of specific tools for bad news communication could exacerbate stressful conditions at this sensitive time among healthcare providers. The present findings could be used by health institutions to develop ad hoc training programs for both physicians and nurses, as well as to strengthen their organizational culture.

## 1. Introduction

Communication in healthcare is a key pillar of ensuring quality patient-centered care [1]. It reaches far beyond the exchange of information between healthcare professionals (HCPs) and patients, involving emotional, relational, and cultural aspects. Effective interaction not only contributes to establishing a trusting relationship, but is also instrumental in ensuring correct diagnosis, selection of the most appropriate treatment, and patient adherence to care [2]. Communicating bad news to patients and family members represents one of the most complex challenges for healthcare providers [3]. Bad news can be understood as any information that significantly compromises an individual’s expectations about his or her future [4]. The communication of such news requires great sensitivity, empathy, and competency, as it is a crucial moment in the course of care that can have great impact on the lives of patients and their families. Information about ominous prognoses, which can cause concern and distress, must be delivered with care and caution, using non-traumatizing terminology, accommodating the patient’s fears and without avoiding hopeful elements [5].

Communication of bad news often does not occur effectively because of the lack of specific skills of medical staff in communicating with patients and family members. The main barriers encountered are the use of overly technical language, lack of time, cultural differences, organizational issues, and emotional difficulties [6].

Many nurses, who play a key role in communicating bad news, during and after the treatment process [7], report lacking adequate skills and training to manage patients’ and family members’ reactions. When faced with these challenges, they may experience significant difficulty and consequently engage in avoidant behaviors, distancing themselves from their role as health educators. Therefore, proper training is extremely important to be able to prepare patients to deal with unfavorable diagnoses, to provide emotional support, and to clarify their doubts and concerns related to prognosis. Particularly in oncology and hematology settings, the communication of an adverse diagnosis has a major impact on the patient’s quality of life and the outcome of the disease. In fact, alongside the symptoms of the disease and treatment effects, patients often face profound questions about life’s meaning, suffering, and death.

As with other medical conditions, the suffering associated with receiving a cancer diagnosis can manifest through different channels, including somatic symptoms, sleep disturbances, lack of appetite, and psychiatric disorders [8]. Specific training for and careful recognition of these issues by HCPs could improve their relationship with patients and allow them to provide appropriate emotional support and care [9,10].

To improve clinician–patient communication, various evidence-based models have been created to facilitate the effective communication of bad news. One of the most widely used is the SPIKES model [11]. The acronym considers six key components of the communication process, starting from exploring of the patient’s existing knowledge and expectations to delivering the diagnosis while respecting the patient’s pace and preferences. Specifically: Setting (S) refers to preparing the environment for the interview; Perception (P) involves assessing what the patient already understands; Invitation (I) concerns determining how much information the patient wants to receive; Knowledge (K) refers to sharing information with the patient; Emotions (E) involves recognizing and responding to the patient’s reactions; and Summary (S) focuses on outlining the next steps and summarizing the main points discussed. This model was designed to improve communication outcomes in difficult contexts such as advanced cancer or palliative or end-of-life settings [12]. It allows for a gradual and structured approach, providing information progressively to avoid overwhelming the patient and increasing anxiety. Its application is flexible, takes the content into consideration, and can be adapted to the patient’s clinical and psychosocial conditions.

In a previous study, the higher participation of nurses compared with physicians highlighted the need to overcome the belief that communicating bad news is exclusively the physician’s responsibility. However, the same study found that a high proportion of HCPs had acquired their communication skills solely through work experience [13]. To date, only a few studies in the oncology and hematology fields in Italy have addressed this topic.

The present study aimed to explore the relationship between levels of perceived stress and HCPs’ knowledge, self-reported skills and methods applied when approaching bad news communication. It also sought to investigate the difficulties experienced by HCPs and the emotions they observe in patients during the delivery of bad news in both oncology and hematology settings.

## 2. Materials and Methods

This was a cross-sectional observational study, carried out from October 2023 to September 2024, through the administration of an online questionnaire addressed to both physicians and nurses employed in oncology and hematology health services in Italy.

The survey tool was created using the Google forms platform and shared through the Italian Group for Bone Marrow Transplantation (GITMO) network and the virtual area of “Noi delle Cure Palliative” that agreed to participate in the present study.

The questionnaire was the same as that used in our previous research involving physicians and nurses working in all healthcare settings [13]. In this new study, we administered the questionnaire exclusively to physicians and nurses employed in oncological and hematological services across Italy, regardless of care setting (inpatient or outpatient), patient age (adult or pediatric), or disease stage (palliative or active treatment).

The first part of the questionnaire collected demographic characteristics, such as sex, civil status, religion, work experience in the oncology field, educational level, oncology setting, and job role.

The second section of the questionnaire included items investigating participants’ self-perceived knowledge of evidence-based methods (e.g., SPIKES) used during the bad news communication process, as well as items regarding workplace resources available for supporting bad news communication in cancer care.

The third part of the questionnaire included items exploring self-perceived difficulties met in managing the bad news communication process. The fourth section consisted of items assessing the skills and competences applied by HCPs during the communication, as well as the emotional reactions observed in patients during or immediately after the delivery of bad news. To assess knowledge, difficulties, skills, and patients’ feelings, the questionnaire provided three response options (“never”, “sometimes”, “often”). The final part of the questionnaire included the “Perceived Stress Scale (PSS)” [14], which represents the most widely used psychological tool for the self-assessment of perceived stress. The scale includes 10 items that assess different dimensions of perceived stress using a five-point Likert scale, where “0” indicates “never” and “4” indicates “very often”. Participants were asked to rate how often they felt in the situation indicated by each item. By summing all item responses, a total score was obtained, ranging from low perceived stress (total score: 0–13), to moderate perceived stress (total score: 14–26), and high perceived stress (total score: 27–40). The PSS has demonstrated good psychometric properties (Cronbach α > 0.70; test–retest > 0.70) [15].

A total of 508 professionals received the study proposal through the participating networks’ communication channels (emails and web platforms). This corresponds to approximately 10% of all physicians and nurses working in oncology settings in Italy [16]. Considering a 95% confidence level, a 5% margin of error, and an assumed standard deviation of 50%, a required sample size of 219 participants was calculated using Cochran’s formula. Data were downloaded into an Excel^®^ spreadsheet [version 16.0, 2021. Microsoft Corporation, Redmond, WA 98052-6399, USA]. Socio-demographic characteristics and the findings of the “ad hoc” questionnaire were described using frequencies and percentages.

To compare the PSS score levels with the questionnaire findings, respondents were clustered into 3 groups according to their PSS score: the “Low Stress” group (score = 0–13), the “Moderate Stress” group (score = 14–26), and the “High Stress” group (score = 27–40). Multiple comparison chi-square tests and generalized Fisher’s exact tests were used to identify significant differences among three or more percentages for unpaired data. Fisher’s exact test was used in cases where the assumptions for the chi-square test were not met. Additionally, we performed a power analysis for each statistical test based on the corresponding effect size. Effect sizes were calculated using the phi coefficient for categorical variables, η2 and r for non-parametric test (Mann–Whitney test and Wilcoxon signed-rank test, respectively), and Cohen’s for paired and unpaired *t*-tests. Statistical significance was set at a threshold of *p* < 0.05. All analyses were performed using the MATLAB (Matrix Laboratory) analytical toolbox 2008 (MathWorks, Natick, MA, USA) for Windows at 32 bits.

The present study was approved by both the GITMO trial office and the board of “Noi delle Cure Palliative” in January 2024, which disseminated the questionnaire link to all Italian nurses and physicians registered in their networks. To protect participants’ privacy, the questionnaire was completely anonymous; no contact details, location, or workplace were collected, and age was recorded only in aggregated groups. Therefore, in accordance with the principles of the General Data Protection Authority (GDPR—EU 2026/679) and Italian privacy regulations, ethical committee authorization was not required.

The study aims and objectives were described in a cover letter, which also emphasized the voluntary nature of participation. Only participants who provided consent by checking a specific box were able to complete the questionnaire.

## 3. Results

A total of 221 Italian HCPs completed the questionnaire, corresponding to 43.5% of the invited population. Of these, 68 (30.8%) were physicians and 153 (69.2%) nurses, all employed in oncology or hematology settings (Table 1). Of these, 118 (53.4%) were female and 103 (46.6%) were male; 116 (52.5%) were married and most (154; 69.7%) identified as Christian. Nearly half of the participants (102; 46.2%) had worked in oncology settings for less than 5 years, and 115 (52%) declared to have bachelor’s degree. Participants were employed in hematology settings (93; 42.1%), oncology (50; 22.6%), or mixed oncology–hematology centers (17; 7.7%). In addition, 33 (14.9%) worked in pediatric oncology–hematology centers, and 28 (12.7%) in palliative care units.

Regarding knowledge and use of bad news communication methods (Table 2), the majority of participants (135; 61.1%) reported having learned to conduct difficult conversations from a mentor during clinical practice, while 125 (56.6%) had attended at least one course on communication techniques. Although most participants reported good or very good self-perceived communication competence and relationship skills (126; 57.0%, and 149; 67.4%, respectively), the large majority acknowledged experiencing difficulties during interviews with patients and their families (186; 84.2%). Moreover, more than half of the participants felt they were not very competent in managing difficult conversations (128; 57.9%). However, 141 participants (63.8%) reported not knowing the SPIKES method. Most participants considered the way patients are welcomed to be very important in their daily work (119; 53.8%), and 190 (86.0%) regarded the time dedicated to communication as important or very important for patient recovery. Nevertheless, 154 participants (69.7%) stated that they did not have dedicated spaces for delivering bad news, which most often took place in the physician’s office or the patient’s room (Table 2).

When exploring the self-perceived levels of stress experienced by HCPs when approaching bad news communication, we found that the majority of participants reported “often” experiencing a moderate level of stress across all items considered (Table 3). Significant differences in response distribution according to PSS score were found for the following items: “communicating the truth” (*p* = 0.010), “preparing the interview” (*p* = 0.001), “structuring the message” (*p* < 0.001), “proposing the message and intervention” (*p* = 0.001 and *p* = 0.015), and “maintaining congruence between speech and body language” (*p* < 0.001). Power analysis indicated a large effect size for all statistically significant tests, suggesting a reduced likelihood of statistical bias. All non-significant tests showed a medium effect size (Table 3).

Similarly, many participants who completed the section on attitudes and competences related to bad news communication reported “often” experiencing moderate stress across all investigated items (Table 4). In particular, a significant proportion of respondents (87; 39.4%) indicated feeling this way when introducing themselves during the initial approach (*p* = 0.006), and 98 (44.3%) reported similar stress when attempting to anticipate which psychosocial areas might become imbalanced following the delivery of bad news (*p* = 0.044). Additionally, most participants reported “often” experiencing moderate stress when the patient refused to become informed (88, 39.8%; *p* = 0.006), when they were required to appear assertive and confident regardless of the patient’s reaction (87, 39.4%; *p* = 0.013), and when monitoring the patient’s reactions after the delivery of bad news (126, 57.0%; *p* = 0.012). Significant differences between groups were also found for these items. One hundred participants (45.2%) reported “sometimes” experiencing moderate stress when planning the timing of the communication. As reported in Table 4, a large effect size was found for all significant tests, indicating a reduced likelihood of statistical bias. Many non-significant tests showed a medium effect size, while two non-significant tests showed a large effect size (“Do you consider the patient’s opinion?” and “Do you maintain an active listening attitude regardless of the patient’s reaction?”). This discrepancy between effect size magnitude and statistical significance may be due to the limited sample size or high variability within the data. In any case, differences in group size maybe have influenced the results, which should be further investigated in studies with larger samples.

Cross-tables among participants’ PSS levels and the frequencies of patients’ emotions observed after the communication process were performed (Table 5). Most participants who reported “often” observing certain emotional reactions in patients also showed moderate levels of perceived stress. This was particularly evident for feelings of personal failure (94; 42.5%), despair (99; 44.8%), discouragement (90; 40.7%), lack of motivation (68; 30.8%), loss of life-purpose (74; 33.5%), depression (116; 52.5%), loss of interest (83; 37.5%), weight loss (83; 37.5%), sleep disorders (91; 41.2%), and psychomotor agitation (130; 58.8%). Conversely, moderate stress levels did not appear to be associated with the observation of other emotional responses, such as feelings of personal devaluation, suicidal ideation, reduced attention, social isolation, a sense of entrapment, or “freezing”, or behaviors conflicting with social norms.

Significant differences in group distributions were identified for several patient emotions, including feelings of personal failure (*p* = 0.002), despair (*p* = 0.003), discouragement (*p* < 0.001), lack of motivation (*p* < 0.001), loss of life purpose (*p* = 0.010) loss of interest (*p* < 0.001), decreased attention (*p* < 0.001), social isolation (*p* = 0.004), sense of entrapment (*p* < 0.001), behaviors against morality (*p* = 0.048), and sense of freezing (*p* = 0.004). One fifth of participants (45; 20.4%) reported observing suicidal ideation “sometimes” or “often” in patients after the communication of bad news. Power analysis provided results similar to those obtained in Table 3 and Table 4. Particularly, all significant tests showed a large effect size, meaning that the significant statistical tests showed a reduced presence of statistical bias. All non-significant tests showed a medium effect size.

## 4. Discussion

The present research aimed to evaluate approaches to bad news communication and the related difficulties encountered by HCPs working in oncology and hematology settings. Specifically, we explored their attitudes and knowledge regarding the communication of bad news across all stages of the communication process. We then explored the significant associations between PSS scores and questionnaire responses.

Our study sought to explore the relationship between HCPs’ perceived stress and the frequency with which they observed patients’ emotional reactions following the delivery of bad news. However, the vast majority of participants reported moderate stress levels across all emotional categories, resulting in substantial differences in group size. This imbalance limited our ability to draw reliable conclusions about the association between perceived stress and the frequency of observed emotional reactions. Nevertheless, our results show that HCPs frequently observed a series of common emotional reactions in patients receiving bad news, including feelings of failure, despair, discouragement, lack of motivation or life purpose, depression, decreased interest, weight loss, and sleep disturbances. Although these reactions may be influenced by patients’ personality traits and life experiences, they can be effectively addressed when physicians and nurses collaborate with specialized professionals such as psychologists, occupational therapists, and social workers.

Less frequent but more concerning were findings of self-harming ideation, the adoption of immoral behaviors, loneliness, personal devaluation, and a sense of entrapment (Table 5). These manifestations may reflect deeper emotional distress and could have serious consequences for patients’ life trajectories. Thus, they should be promptly recognized by oncology HCPs, and adequately assessed by mental health specialists [17].

Our findings highlight that many HCPs experience difficulties and moderate levels of stress when communicating bad news to patients. In addition, though they reported having good skills in managing difficult discussions, they often perceived themselves as poorly competent, did not apply guidance such as the SPIKES method, and reported difficulties across all phases of the communication process. In Italy, several factors may hinder the patient’s right to autonomy during the delivery of bad news. Among the cultural factors, traditional medical paternalism often leads clinicians to prioritize emotional protection over full disclosure, with the intention of avoiding patient distress. Likewise, the central role of the family in Italian society results in filtering or mediating health information, placing greater emphasis on preserving the patient’s composure and tranquility rather than safeguarding their right to be fully informed. In this context, HCPs may adopt a diplomatic and indirect approach, relying on allusions or implicit communication, particularly when conveying unfavorable or difficult news. However, intergenerational differences must also be taken into account, as younger generations increasingly reject concealment of the truth, driving a cultural shift toward greater autonomy and transparency [18,19,20,21,22]. These factors, combined with training gaps and organizational limitations, which we will discuss below, may have increased HCPs’ discomfort contributed to the tendency to avoid fully transparent disclosure. This situation created a significant discrepancy in our study, particularly regarding the gap between the number of trained practitioners, and both their knowledge of the SPIKES method and own perception of competence. Possible explanations include the limited efficacy of the available educational programs, insufficient engagement from HCPs, or the absence of robust systems for ongoing communication skills maintenance [23].

Additionally, a limited availability of dedicated spaces within workplaces was reported, with difficult conversations most often occurring in the physician’s office or in the patient’s room. This finding may reflect cultural and organizational limitations within Italian health institutions, which often do not consider the delivery of bad news as fundamental components of the oncology care pathways, nor fully recognize the burden it places on HCPs. The lack of private, dedicated environments highlights insufficient institutional attention to the dynamics of this practice, leaving HCPs to assume full responsibility for this conversation under suboptimal and stressful conditions. This disproportionate delegation of responsibility to both physicians and nurses is indicative of elevated stress levels and a lack of institutional acknowledgment of the complexity inherent in this type of communication [12,24].

The implementation of robust organizational support systems for HCPs who routinely manage individuals with life-limiting illnesses could potentially alleviate the distress associated with challenging communication tasks and improve patient satisfaction [25].

Our results describe a concerning scenario that must be addressed by health institutions, starting with the provision of supportive organizational environments, continuous education, and greater engagement of HCPs [26]. Adequate communication competencies should be considered a fundamental requirement for HCP qualification [27], and fostering improved attitudes among healthcare professionals, particularly regarding self-awareness, reflection, and the continuous development of their communication skills, could promote better outcomes for both patients and HCPs. In this direction, further studies are needed to better investigate existing training gaps in bad news communication [2].

Comparing our results with the existing literature, several additional considerations emerge. Strategies for learning and acquiring skills in bad news communication have been widely debated in healthcare worldwide [28,29,30], both in terms of training methods and perceptions of their effectiveness [29,31]. Although several educational programs have demonstrated short-term benefits in improving HCP communication skills, the literature has not been able to demonstrate which types of learning interventions are most effective, nor how long-lasting their effects may be over time [29]. In addition, no robust evidence is currently available regarding the impact of such programs on reducing HCP burnout or improving patient satisfaction [2].

The on-field experiential model remains the preferred training approach for both physicians and nurses to acquire communication skills. However, this method is associated with considerable variability in behaviors, including incorrect or maladaptive ones, and carries the risk of errors during the learning phase [2,32]. More advanced training strategies have recently been exploring, incorporating the effects of stress arousal and integrating evidence-based method such as SPIKES protocol, as well as multimedia, step-by-step learning programs. These approaches aim to emphasize the role of stress coping in improving the effectiveness of difficult conversations [33,34,35]. Preliminary evidence also suggest that conversational artificial intelligence may enhance learners’ confidence in delivering difficult news, offering an additional innovative tool in communication skills training [36].

With the increasing use of web-based learning tools, newer generations of HCPs consider these platforms as particularly effective for their education. This trend is promoting the adoption of multimodal learning methods that allow trainees to acquire adequate skills through evidence-based pathways [2]. However, although foundational training in communication principles provides an essential starting point, these skills must be continuously refined through practical experience with real patients. Establishing robust relational support systems for HCPs (e.g., the availability of a psychologist) may further benefit the practitioners, improve the quality of patient care and satisfaction, and potentially mitigate the incidence of burnout [37].

We found a relationship between higher levels of perceived stress levels and greater difficulty in using evidence-based structured methods (such as SPIKES) when breaking bad news. In addition to the considerations discussed above, this outcome should be carefully considered by health institutions, as heightened stress may lead HCPs to adopt maladaptive communication behaviors, potentially compromising patient’s quality of life during and after treatment [33].

For example, the literature reports that while most HCPs address emotional issues during their interviews with cancer patients [38,39], the time dedicated to biomedical discussions remains predominant compared with that devoted to psychosocial issues [40]. This imbalance may both contribute to and result from the difficulties in communicating the truth and managing the delivery of difficult news observed in our sample (Table 3). Moreover, it is essential to consider that ineffective communication and increased stress are associated with reduced job satisfaction and a higher risk of emotional burnout among HCPs [41,42].

Our study highlights the need for deeper reflection on HCPs’ self-awareness and on their ability to learn skills relevant to the communication process in order to prevent burnout and enhance patient outcomes. In line with previous authors, we believe that the effectiveness of training programs aimed at improving bad news delivery skills will remain a major challenge as long as the relationship between HCP stress and patients’ emotional response continues to be undervalued [43,44].

Some authors have explored the effects of personalized training programs aimed at improving patients’ communication skills prior to medical interviews, suggesting that such interventions may improve the efficacy of learning programs designed for HCPs as well [45,46]. However, the relationship between patient and HCP depends on various factors beyond individual skills [47], including cultural context, environmental conditions, and family influences. As a result, communication dynamics may vary substantially, regardless of the intentions of the stakeholders involved [38,39,48]. In this regard, a thorough understanding of each patient’s needs is essential to prevent misunderstandings and potential litigation [49,50].

### Strengths and Limitations

The present study provides a snapshot of current practices in bad news communication within oncology and hematology settings in Italy, highlighting the difficulties experienced by both nurses and physicians when delivering unfavorable information to patients. The study involved a large cohort of participants, without considering various factors that could impact the results. The relationship between HCPs stress levels and the variables chosen to describe their approach to the bad news delivery process was assessed; however, differences among group sizes limited the generalizability of our results. The sample included both physicians and nurses, who have different responsibilities in managing communication with the patients; this may have influenced different perceptions of the questionnaire’s importance. A selection bias may have introduced by the recruitment method, which involved only nurses and physicians belonging to available voluntary groups. The topic of the questionnaire and its length may have selected participants who were particularly motivated to participate (self-selection bias), and this might have increased both social desirability bias and question-order bias. For these reasons, the study findings should be considered with caution, as they may not fully represent the entire population.

## 5. Conclusions

The present study highlighted many characteristics of bad news communication between HCPs (physicians and nurses) and their oncology patients. The association between the responses provided and the stress levels perceived by participants during and after the bad news delivery process further emphasized the stressful conditions experienced by providers at this sensitive time. Considering communications skills as mandatory training components for proving adequate care to cancer patients, these findings may offer useful data on the current “state of the art” across our country and could be used to inform the implementation of training courses and development of new strategies for competence acquisition and maintenance. However, changes in the organizational culture of the Italian Health Services would be desirable in order to allow for the effective application of the skills acquired by HCPs during training, to limit maladaptive behaviors, and to improve patient satisfaction.

## Figures and Tables

**Table 1:** Sample socio-demographic characteristics (n = 221).

Items	Characteristics	n (%)
Sex	Female	118 (53.4)
	Male	103 (46.6)
Civil status	Unmarried	61 (27.6)
	Married	116 (52.5)
	Divorced/Separated	38 (17.2)
	Widower	6 (2.7)
Religion	Christian	154 (69.7)
	Atheist	37 (16.7)
	Agnostic	27 (12.2)
	Other	3 (1.40)
Work experience in oncology	1–5 years	102 (46.2)
	6–15 years	61 (27.6)
	16–25 years	24 (10.8)
	>25 years	34 (15.4)
Higher academic level	Bachelor’s degree	115 (52.0)
	Master’s degree	33 (14.9)
	Degree in Medicine	7 (3.2)
	PhD	61 (27.6)
	Other	5 (2.3)
Setting	Hematology inpatient	49 (22.2)
	Hematology outpatient	26 (11.8)
	Oncology inpatient	33 (14.9)
	Oncology outpatient	17 (7.7)
	HSCT	18 (8.1)
	Pediatric oncology–hematology inpatient	33 (14.9)
	Mixed oncology–hematology inpatient	17 (7.7)
	Palliative care unit	28 (12.7)
Job role	Physician	68 (30.8)
	Nurse	153 (69.2)

HSCT = Hematopoietic stem cell transplantation.

**Table 2:** Self-reported knowledge of, and application of bad news communication methods (n = 221).

Questions		Items	n (%)
Have you ever met someone who taught you how to communicate bad news to patient and family?		Yes	135 (61.1)
		No	86 (38.9)
Have you ever attended training courses on bad news communication techniques?		Yes	125 (56.6)
		No	96 (43.4)
Do you know the SPIKES method?		Yes	80 (36.2)
		No	141 (63.8)
How do you rate your competence level in difficult talking management?		Poor	9 (4.1)
		Moderate	119 (53.8)
		Good	78 (35.3)
		Very good	15 (6.8)
How do you rate your communication skills during difficult talking?		Poor	40 (18.1)
		Moderate	55 (24.9)
		Good	95 (43.0)
		Very good	31 (14.0)
How do you rate your ability to establish and maintain the relationship with both the patient and its family?		Poor	21 (9.5)
		Moderate	51 (23.1)
		Good	110 (49.8)
		Very good	39 (17.6)
How do you rate your confidence having difficult talking with the patient and its family?		Poor	9 (4.1)
		Moderate	26 (11.8)
		Good	123 (55.7)
		Very good	63 (28.5)
During working hours, what extent do you feel it is important to welcome patients?		Not at all	42 (19.0)
		Slightly	39 (17.6)
		Important	60 (27.1)
		Very	119 (53.8)
What extent do you consider the time spent with a patient important for his/her recovery?		Not at all	1 (0.5)
		Slightly	30 (13.6)
		Important	91 (41.2)
		Very	99 (44.8)
In your center, are there dedicated spaces for bad news communication?		Yes	67 (30.3)
		No	154 (69.7)
In your center, where does bad news communication may occur?	Patient’s room	Never	42 (19.2)
		Rarely	27 (12.2)
		Often	76 (34.4)
		Always	76 (34.4)
	Physician’s room	Never	27 (12.2)
		Rarely	34 (15.4)
		Often	72 (32.6)
		Always	88 (39.8)
	Unprotected space (e.g., lobby, hallway)	Never	183 (82.3)
		Rarely	29 (13.1)
		Often	7 (3.2)
		Always	2 (0.9)
	Dedicated space	Never	166 (75.1)
		Rarely	8 (3.6)
		Often	17 (7.7)
		Always	30 (13.6)

**Table 3:** Difficulties experienced by the participants in managing bad news communication process according to PSS scoring levels (n = 221).

Items	Responses	PSS Score n (%)			*p*-Value (Test)	Effect Size
		Low (0–13)	Moderate (14–26)	High (27–40)		
Communicating the truth	Never	11 (5.0)	33 (14.9)	4 (1.8)	0.010 (F) *	Phi = 1.05 Large effect
	Sometimes	18 (8.1)	109 (49.3)	10 (4.5)		
	Often	5 (2.3)	21 (9.5)	10 (4.5)		
Applying the SPIKES method	Never	19 (8.6)	58 (26.2)	9 (4.1)	0.147 (C)	Phi = 0.46 Medium effect
	Sometimes	9 (4.1)	68 (30.8)	7 (3.2)		
	Often	6 (2.7)	37 (16.7)	8 (3.6)		
Prepare the interview	Never	22 (10.0)	43 (19.5)	6 (2.7)	0.001 (F) *	Phi = 1.32 Large effect
	Sometimes	11 (5.0)	109 (49.3)	16 (7.2)		
	Often	1 (0.5)	11 (5.0)	2 (0.9)		
Structuring the message	Never	24 (10.9)	42 (19.0)	6 (2.7)	<0.001 (F) *	Phi = 1.92 Large effect
	Sometimes	10 (4.5)	111 (50.2)	15 (6.8)		
	Often	0 (0)	10 (4.5)	3 (1.4)		
Propose the message	Never	22 (10.0)	45 (20.4)	5 (2.3)	0.001 (F) *	Phi = 1.35 Large effect
	Sometimes	12 (5.4)	108 (48.9)	17 (7.7)		
	Often	0 (0)	10 (4.5)	2 (0.9)		
Intervention proposal	Never	18 (8.1)	40 (18.1)	4 (1.8)	0.015 (F) *	Phi = 0.88 Large effect
	Sometimes	14 (6.3)	106 (48)	17 (7.7)		
	Often	2 (0.9)	17 (7.7)	3 (1.4)		
Maintain congruence between speech and body languages	Never	21 (9.5)	49 (22.2)	5 (2.3)	<0.001 (F) *	Phi = 1.75 Large effect
	Sometimes	12 (5.4)	108 (48.9)	14 (6.3)		
	Often	1 (0.5)	6 (2.7)	5 (2.3)		
Communicating bad news to a very young patient	Never	5 (2.3)	12 (5.4)	1 (0.5)	0.092 (F)	Phi = 0.53 Medium effect
	Sometimes	19 (8.6)	85 (38.5)	8 (3.6)		
	Often	10 (4.5)	66 (29.9)	15 (6.8)		

PSS = Perceived Stress Score. * *p* < 0.05 significant difference. C = chi-square test, F = generalized Fisher’s exact test.

**Table 4:** Self-reported skills on bad news communication according to PSS scoring levels (n = 221).

Items (Questions)	Responses	PSS Score n (%)			*p*-Value (Test)	Effect Size
		Low (0–13)	Moderate (14–26)	High (27–40)		
Do you choose a quiet and confidential place for bad news communication?	Never	3 (1.4)	5 (2.3)	1 (0.5)	0.370 (F)	Phi = 0.25 Medium effect
	Sometimes	17 (7.7)	78 (35.3)	9 (4.1)		
	Often	14 (6.3)	80 (36.2)	14 (6.3)		
Do you make sure there will be no interruption? (by phone, colleagues, etc.)	Never	3 (1.4)	6 (2.7)	1 (0.5)	0.510 (F)	Phi = 0.19 Low effect
	Sometimes	16 (7.2)	86 (38.9)	10 (4.5)		
	Often	15 (6.8)	71 (32.1)	13 (5.9)		
Do you plan the communication time?	Never	13 (5.9)	34 (15.4)	7 (3.2)	0.006 (C) *	Phi = 0.98 Large effect
	Sometimes	9 (4.1)	100 (45.2)	11 (5)		
	Often	12 (5.4)	29 (13.1)	6 (2.7)		
Do you introduce yourself first of all?	Never	7 (3.2)	5 (2.3)	0 (0)	0.006 (F) *	Phi = 1.32 Large effect
	Sometimes	12 (5.4)	71 (32.1)	8 (3.6)		
	Often	15 (6.8)	87 (39.4)	16 (7.2)		
Do you use his first name (talking to patient)?	Never	4 (1.8)	9 (4.1)	1 (0.5)	0.590 (F)	Phi = 0.19 Low effect
	Sometimes	12 (5.4)	75 (33.9)	11 (5)		
	Often	18 (8.1)	79 (35.7)	12 (5.4)		
Do you look his face/eyes (talking to patient)?	Never	3 (1.4)	9 (4.1)	1 (0.5)	0.820 (F)	Phi = 0.10 Low effect
	Sometimes	14 (6.3)	69 (31.2)	8 (3.6)		
	Often	17 (7.7)	85 (38.5)	15 (6.8)		
Before starting the interview, do you promote the participation of a relative if authorized by the patient?	Never	8 (3.6)	25 (11.3)	3 (1.4)	0.780 (F)	Phi = 0.12 Low effect
	Sometimes	20 (9)	103 (46.6)	15 (6.8)		
	Often	6 (2.7)	35 (15.8)	6 (2.7)		
Before starting the interview, do you try to know what the patient may have intuited about his condition?	Never	7 (3.2)	19 (8.6)	1 (0.5)	0.450 (F)	Phi = 0.26 Medium effect
	Sometimes	19 (8.6)	99 (44.8)	15 (6.8)		
	Often	8 (3.6)	45 (20.4)	8 (3.6)		
Do you try to anticipate the understanding of which psychosocial areas may result imbalanced by the bad news?	Never	10 (4.5)	21 (9.5)	1 (0.5)	0.044 (F) *	Phi = 0.72 Large effect
	Sometimes	8 (3.6)	44 (19.9)	4 (1.8)		
	Often	16 (7.2)	98 (44.3)	19 (8.6)		
If the patient doesn’t want to be informed, do you give him the time he needs to think about it?	Never	7 (3.2)	17 (7.7)	0 (0)	0.006 (F) *	Phi = 0.94 Large effect
	Sometimes	8 (3.6)	58 (26.2)	3 (1.4)		
	Often	19 (8.6)	88 (39.8)	21 (9.5)		
Do you promote the expression of patient’s emotions?	Never	6 (2.7)	14 (6.3)	0 (0)	0.180 (F)	Phi = 0.43 Medium effect
	Sometimes	11 (5.0)	45 (20.4)	7 (3.2)		
	Often	17 (7.7)	104 (47.1)	17 (7.7)		
Do you consider the patient’s opinion?	Never	7 (3.2)	13 (5.9)	0 (0)	0.059 (F)	Phi = 0.66 Large effect
	Sometimes	5 (2.3)	35 (15.8)	8 (3.6)		
	Often	22 (10.0)	115 (52.0)	16 (7.2)		
Do you promote the expression of patient’s point of view about the situation?	Never	4 (1.8)	11 (5.0)	0 (0)	0.120 (F)	Phi = 0.49 Medium effect
	Sometimes	11 (5.0)	43 (19.5)	3 (1.4)		
	Often	19 (8.6)	109 (49.3)	21 (9.5)		
Do you use a clear language that facilitate patient’s understanding?	Never	4 (1.8)	9 (4.1)	0 (0)	0.230 (F)	Phi = 0.40 Medium effect
	Sometimes	11 (5.0)	55 (24.9)	5 (2.3)		
	Often	19 (8.6)	99 (44.8)	19 (8.6)		
Do you give information in a sequential and organized manner?	Never	5 (2.3)	13 (5.9)	2 (0.9)	0.360 (F)	Phi = 0.28 Medium effect
	Sometimes	9 (4.1)	44 (19.9)	3 (1.4)		
	Often	20 (9.0)	106 (48.0)	19 (8.6)		
Do you ask to the patient what his feelings are?	Never	7 (3.2)	13 (5.9)	1 (0.5)	0.210 (F)	Phi = 0.45 Medium effect
	Sometimes	7 (3.2)	31 (14.0)	4 (1.8)		
	Often	20 (9.0)	119 (53.8)	19 (8.6)		
Do you maintain an active listening attitude regardless of the patient’s reaction?	Never	7 (3.2)	9 (4.1)	2 (0.9)	0.058 (F)	Phi = 0.66 Large effect
	Sometimes	9 (4.1)	42 (19.0)	4 (1.8)		
	Often	18 (8.1)	112 (50.7)	18 (8.1)		
Do you show a nonverbal attitude of support and understanding?	Never	6 (2.7)	10 (4.5)	2 (0.9)	0.290 (F)	Phi = 0.35 Medium effect
	Sometimes	9 (4.1)	44 (19.9)	6 (2.7)		
	Often	19 (8.6)	109 (49.3)	16 (7.2)		
Do you behave assertively, expressing your thoughts confidently?	Never	8 (3.6)	10 (4.5)	1 (0.5)	0.013 (F) *	Phi = 0.95 Large effect
	Sometimes	9 (4.1)	66 (29.9)	5 (2.3)		
	Often	17 (7.7)	87 (39.4)	18 (8.1)		
In case of patient’s disagreement with a proposed treatment, do you discuss with him alternative ways?	Never	4 (1.8)	11 (5.0)	1 (0.5)	0.570 (F)	Phi = 0.19 Low effect
	Sometimes	8 (3.6)	35 (15.8)	3 (1.4)		
	Often	22 (10.0)	117 (52.9)	20 (9.0)		
Do you monitor the feelings expressed by the patient after the bad news?	Never	6 (2.7)	8 (3.6)	0 (0)	0.012 (F) *	Phi = 0.95 Large effect
	Sometimes	9 (4.1)	29 (13.1)	2 (0.9)		
	Often	19 (8.6)	126 (57.0)	22 (10.0)		
Do you check that the patient has no residual doubts after the interview?	Never	7 (3.2)	13 (5.9)	1 (0.5)	0.260 (F)	Phi = 0.42 Medium effect
	Sometimes	7 (3.2)	42 (19.0)	6 (2.7)		
	Often	20 (9.0)	108 (48.9)	17 (7.7)		
Do you plan strategies to improve patients’ coping?	Never	4 (1.8)	16 (7.2)	1 (0.5)	0.540 (F)	Phi = 0.24 Medium effect
	Sometimes	7 (3.2)	41 (18.6)	3 (1.4)		
	Often	23 (10.4)	106 (48.0)	20 (9.0)		

PSS = Perceived Stress Score. * *p* < 0.05 significant difference. C = chi-square test, F = generalized Fisher’s exact test.

**Table 5:** Participants’ PSS levels and frequencies of observed patients’ emotions (n = 221).

Patients’ Feelings	Responses	Overall	PSS Score n (%)			*p*-Value (Test)	Effect Size
			Low (0–13)	Moderate (14–26)	High (27–40)		
Feeling of personal failure	Never	41 (18.6)	12 (5.4)	27 (12.2)	2 (0.9)	0.002 (F) *	Phi = 1.09 Large effect
	Sometimes	68 (30.8)	12 (5.4)	55 (24.9)	1 (0.5)		
	Often	112 (50.6)	8 (3.6)	94 (42.5)	10 (4.6)		
Despair	Never	25 (11.3)	8 (3.6)	16 (7.2)	1 (0.5)	0.003 (F) *	Phi = 1.04 Large effect
	Sometimes	65 (29.4)	14 (6.3)	48 (21.7)	3 (1.4)		
	Often	131 (59.3)	12 (5.4)	99 (44.8)	20 (9.1)		
Discouragement	Never	43 (19.5)	10 (4.6)	33 (14.8)	0 (0)	<0.001(F) *	Phi = 1.71 Large effect
	Sometimes	55 (24.9)	14 (6.3)	40 (18.1)	1 (0.5)		
	Often	123 (55.6)	10 (4.6)	90 (40.7)	23 (10.4)		
Motivation lacking	Never	51 (23.1)	14 (6.3)	36 (16.3)	1 (0.5)	<0.001 (C)*	Phi = 1.52 Large effect
	Sometimes	76 (34.4)	13 (5.9)	59 (26.7)	4 (1.8)		
	Often	94 (42.5)	7 (3.1)	68 (30.8)	19 (8.6)		
life-purpose lacking	Never	60 (27.1)	12 (5.4)	45 (20.4)	3 (1.4)	0.010 (C) *	Phi = 0.89 Large effect
	Sometimes	64 (29.0)	15 (6.8)	44 (19.9)	5 (2.3)		
	Often	97 (43.9)	7 (3.1)	74 (33.5)	16 (7.2)		
Depression	Never	16 (7.2)	4 (1.8)	12 (5.4)	0 (0)	0.270 (F)	Phi = 0.34 Medium effect
	Sometimes	49 (22.2)	10 (4.6)	35 (15.7)	4 (1.8)		
	Often	156 (70.6)	20 (9.1)	116 (52.5)	20 (9.1)		
Loss of interest	Never	41 (18.6)	8 (3.6)	31 (14.0)	2 (0.9)	<0.001 (F) *	Phi = 1.27 Large effect
	Sometimes	68 (30.8)	17 (7.7)	49 (22.2)	2 (0.9)		
	Often	112 (50.6)	9 (4.1)	83 (37.5)	20 (9.1)		
Loss of weight	Never	48 (21.7)	7 (3.1)	35 (15.9)	6 (2.7)	0.490 (C)	Phi = 0.23 Medium effect
	Sometimes	62 (28.1)	13 (5.9)	45 (20.5)	4 (1.8)		
	Often	111 (50.2)	14 (6.3)	83 (37.5)	14 (6.3)		
Sleep disorders	Never	34 (15.4)	4 (1.8)	26 (11.8)	4 (1.8)	0.130 (F)	Phi = 0.45 Medium effect
	Sometimes	61 (27.6)	13 (5.9)	46 (20.8)	2 (0.9)		
	Often	126 (57.0)	17 (7.7)	91 (41.2)	18 (8.1)		
Personal devaluation	Never	97 (43.9)	19 (8.6)	73 (33.0)	5 (2.3)	0.095 (C)	Phi = 0.53 Medium effect
	Sometimes	70 (31.7)	10 (4.5)	50 (22.6)	10 (4.5)		
	Often	54 (24.4)	5 (2.3)	40 (18.1)	9 (4.1)		
Suicide ideations	Never	176 (79.6)	30 (13.6)	130 (58.8)	16 (7.2)	0.280 (F)	Phi = 0.36 Medium effect
	Sometimes	29 (13.1)	3 (1.4)	22 (9.9)	4 (1.8)		
	Often	16 (7.3)	1 (0.5)	11 (5.0)	4 (1.8)		
Attention lacking	Never	121 (54.7)	27 (12.2)	89 (40.2)	5 (2.3)	<0.001 (F) *	Phi = 1.39 Large effect
	Sometimes	59 (26.7)	6 (2.7)	42 (19.0)	11 (5.0)		
	Often	41 (18.6)	1 (0.5)	32 (14.5)	8 (3.6)		
Social isolation	Never	106 (48.0)	23 (10.4)	77 (34.9)	6 (2.7)	0.004 (F) *	Phi = 1.04 Large effect
	Sometimes	62 (28.0)	7 (3.1)	49 (22.2)	6 (2.7)		
	Often	53 (24.0)	4 (1.8)	37 (16.8)	12 (5.4)		
Sense of entrapment	Never	107 (48.4)	24 (10.9)	78 (35.2)	5 (2.3)	<0.001 (C) *	Phi = 1.28 Large effect
	Sometimes	64 (29.0)	9 (4.1)	47 (21.2)	8 (3.6)		
	Often	50 (22.6)	1 (0.5)	38 (17.2)	11 (5.0)		
Behaviors against common morality	Never	147 (66.5)	27 (12.2)	108 (48.9)	12 (5.4)	0.048 (F) *	Phi = 0.74 Large effect
	Sometimes	54 (24.4)	6 (2.7)	42 (19.0)	6 (2.7)		
	Often	20 (9.1)	1 (0.5)	13 (5.9)	6 (2.7)		
Sense of freezing	Never	99 (44.8)	24 (10.9)	70 (31.6)	5 (2.3)	0.004 (C) *	Phi = 1.02 Large effect
	Sometimes	69 (31.2)	6 (2.7)	53 (24.0)	10 (4.5)		
	Often	53 (24.0)	4 (1.8)	40 (18.1)	9 (4.1)		
Psychomotor agitation	Never	13 (5.9)	4 (1.8)	8 (3.6)	1 (0.5)	0.170 (F)	Phi = 0.41 Medium effect
	Sometimes	33 (14.9)	7 (3.1)	25 (11.3)	1 (0.5)		
	Often	175 (79.2)	23 (10.4)	130 (58.8)	22 (10.0)		

PSS = Perceived Stress Score. * *p* < 0.05 significant difference. C = chi-square test, F = generalized Fisher’s exact.

## Data Availability

The original contributions presented in this study are included in the article. Further inquiries can be directed to the corresponding author.

## Public Involvement Statement

No public involvement in any aspect of this research.

## Guidelines and Standards Statement

This manuscript was drafted against the STROBE (Strengthening the Reporting of Observational Studies in Epidemiology) for Cross-Sectional studies.

## Abbreviations

The following abbreviations are used in this manuscript:

HCPs	Healthcare Professionals
SPIKES	Setting Perception Invitation Knowledge Emotions Summary
GITMO	Gruppo Italiano Trapianto di Midollo Osseo
PSS	Perceived Stress Score
GDPA	General Data Protection Authority
HSCT	Hematopoietic Stem Cell Transplantation

## References

[1] Sharkiya S.H. (2023). Quality communication can improve patient-centered health outcomes among older patients: A rapid review. BMC Health Serv. Res..

[2] Moore P.M., Rivera S., Bravo-Soto G.A., Olivares C., Lawrie T.A. (2018). Communication skills training for healthcare professionals working with people who have cancer. Cochrane Database Syst. Rev..

[3] Alshami A., Douedi S., Avila-Ariyoshi A., Alazzawi M., Patel S., Einav S., Surani S., Varon J. (2020). Breaking bad news, a pertinent yet still an overlooked skill: An international survey study. Healthcare.

[4] Rosenzweig M.Q. (2012). Breaking bad news: A guide for effective and empathetic communication. Nurse Pract..

[5] Preti B.T., Sanatani M.S. (2024). Five ways to get a grip on the personal emotional cost of breaking bad news. Can. Med. Educ. J..

[6] Wahyuni S., Gautama M.S.N., Simamora T.Y. (2023). A Literature Review of Nurses Challenges and Barriers in Assisting Patients and Families Facing Breaking Bad News. Indian J. Palliat. Care.

[7] Yazdanparast E., Arasteh A., Ghorbani S., Davoudi M. (2021). The Effectiveness of Communication Skills Training on Nurses’ Skills and Participation in the Breaking Bad News. Iran. J. Nurs. Midwifery Res..

[8] Vitale E., Lupo R., Artioli G., Lezzi A., Secondo D., Mignone A., Calabrò A., Carvello M., Caldararo C., Lezzi P. (2023). How knowledge time influenced anxiety, depression, stress and quality of life levels in patients suffering from Crohn disease: A cross-sectional multicenter study. Acta Biomed..

[9] Carriero M.C., Leo A., Lezzi A., Lupo R., Conte L., Fanizzi A., Massafra R., Vitale E., Carriero A. (2024). Attitudes, Knowledge and Clinical Practice of Health Professionals towards Psychological Disorders in Cancer Patients: An Observational Study. Diseases.

[10] Fernando A., Tokell M., Ishak Y., Love J., Klammer M., Koh M. (2023). Mental health needs in cancer—A call for change. Future Healthc. J..

[11] Buckman R.A. (2005). Breaking bad news: The SPIKES strategy. Community Oncol..

[12] Baile W.F., Buckman R., Lenzi R., Glober G., Beale E.A., Kudelka A.P. (2000). SPIKES-A six-step protocol for delivering bad news: Application to the patient with cancer. Oncologist.

[13] Vitale E., Lupo R., Marra D., D’Abate A., Carvello M., Calabro A., Cucurachi M., Conte L., Botti S., De Mitri O. (2022). Communicating bad news: Attitudes and modes of communication of the health professions. G. Ital. Med. Lav. Ergon..

[14] Cohen S., Kamarck T., Mermelstein R. (1983). A global measure of perceived stress. J. Health Soc. Behav..

[15] Lee E.H. (2012). Review of the psychometric evidence of the perceived stress scale. Asian Nurs. Res..

[16] Comandone A. (2022). Hospital and primary care setting collaboration: A new model of care in oncology after COVID-19 pandemic. Epidemiol. Prev..

[17] Fujimori M., Hikiji W., Tanifuji T., Suzuki H., Takeshima T., Matsumoto T., Yamauchi T., Kawano K., Fukunaga T. (2017). Characteristics of cancer patients who died by suicide in the Tokyo metropolitan area. Jpn. J. Clin. Oncol..

[18] Berkey F.J., Wiedemer J.P., Vithalani N.D. (2018). Delivering Bad or Life-Altering News. Am. Fam. Physician.

[19] Holmes S.N., Illing J. (2021). Breaking bad news: Tackling cultural dilemmas. BMJ Support. Palliat. Care.

[20] Gordon D.R., Paci E. (1997). Disclosure practices and cultural narratives: Understanding concealment and silence around cancer in Tuscany, Italy. Soc. Sci. Med..

[21] Bongelli R., Bertolazzi A., Piccioni L., Burro R. (2021). Italian onco-haematological patients’ preferences in bad news communication: A preliminary investigation. BMC Cancer.

[22] Costantini A., Baile W.F., Lenzi R., Costantini M., Ziparo V., Marchetti P., Grassi L. (2009). Overcoming cultural barriers to giving bad news: Feasibility of training to promote truth-telling to cancer patients. J. Cancer Educ..

[23] Samuel A., Cervero R.M., Durning S.J., Maggio L.A. (2021). Effect of Continuing Professional Development on Health Professionals’ Performance and Patient Outcomes: A Scoping Review of Knowledge Syntheses. Acad. Med..

[24] Tranberg M., Brodin E.M. (2023). Physicians’ Lived Experience of Breaking Bad News in Clinical Practice: Five Essentials of a Relational Process. Qual. Health Res..

[25] Cerqueira P., Pereira S., Costa R., Sousa B. (2024). Unlocking Team Potential: Mastering Communication in Palliative Care. Cureus.

[26] Cheon J. (2025). End-of-Life Care Stress, Attitudes Toward End-of-Life Care, and End-of-Life Care Performance as Predictors of Job Satisfaction Among Nurses Working in Hospitals in South Korea. Healthcare.

[27] Bylund C.L., Brown R., Gueguen J.A., Diamond C., Bianculli J., Kissane D.W. (2010). The implementation and assessment of a comprehensive communication skills training curriculum for oncologists. Psychooncology.

[28] Weintraub L., Figueiredo L., Roth M., Levy A. (2016). The feasibility of implementing a communication skills training course in pediatric hematology/oncology fellowship. Pediatr. Hematol. Oncol..

[29] Hebert H.D., Butera J.N., Castillo J., Mega A.E. (2009). Are we training our fellows adequately in delivering bad news to patients? A survey of hematology/oncology program directors. J. Palliat. Med..

[30] Kissane D.W., Bylund C.L., Banerjee S.C., Bialer P.A., Levin T.T., Maloney E.K., D’Agostino T.A. (2012). Communication skills training for oncology professionals. J. Clin. Oncol..

[31] Nancekivell S.E., Sun X., Gelman S.A., Shah P. (2021). A Slippery Myth: How Learning Style Beliefs Shape Reasoning about Multimodal Instruction and Related Scientific Evidence. Cogn. Sci..

[32] Lapidow E., Walker C.M. (2022). Rethinking the “gap”: Self-directed learning in cognitive development and scientific reasoning. Wiley Interdiscip. Rev. Cogn. Sci..

[33] Bosshard M., Guttormsen S., Nater U.M., Schmitz F., Gomez P., Berendonk C. (2025). A randomized controlled trial evaluating stress arousal reappraisal and worked example effects on psychophysiological responses during breaking bad news. Sci. Rep..

[34] Bosshard M., Nater U.M., Guttormsen S., Schmitz F., Gomez P., Berendonk C. (2025). Stress arousal reappraisal and worked example effects on the neuroendocrine stress response during breaking bad news in medical education. Psychoneuroendocrinology.

[35] Arumugam K., Nandagopal H., Joseph J., Balaji J.N., Surapaneni K.M. (2024). EMBRACE (Empowering Medical students’ skills in BReaking bAd news with Compassion and Empathy) module improves the skills of undergraduate medical students in effectively breaking the bad news: A case-control study. Adv. Physiol. Educ..

[36] Mukadam A., Suresh S., Jacobs C. (2025). Beyond Traditional Simulation: An Exploratory Study on the Effectiveness and Acceptability of ChatGPT-4o Advanced Voice Mode for Communication Skills Practice Among Medical Students. Cureus.

[37] Gohal A.Y.A., Hakami K.I.H., Al Anazi M.A.K., Alarjani F.M., Bahkali H.J., Aljuhani A.B., Ashamlani K.S., Al Sabar Y.I., Al-Anazi S.K., Hatroosh W.A.M. (2023). Enhancing Nurses’ Well-Being and Performance: The Role of Support Mechanisms in Mitigating Burnout and Improving Healthcare Outcomes. Rev. Contemp. Philos..

[38] Taylor J.S. (2011). The moral aesthetics of simulated suffering in standardized patient performances. Cult. Med. Psychiatry.

[39] Ruiz Sancho E., Pérez Nieto M.Á., Román F.J., León Mateos L., Sánchez Escamilla F., Enrech Francés S., Pérez Escutia M.Á., Juez Mertel I., Pérez-Segura P., Aguirre Herrero A. (2024). Differences in the Communication of Cancer Diagnoses by Different Health Professionals and the Impact of Oncologist Communication on Patients’ Emotions. Cancers.

[40] Hack T.F., Ruether J.D., Pickles T., Bultz B.D., Chateau D., Degner L.F. (2012). Behind closed doors II: Systematic analysis of prostate cancer patients’ primary treatment consultations with radiation oncologists and predictors of satisfaction with communication. Psychooncology.

[41] Fallowfield L.J. (2000). How to improve the communication skills of oncologists. Ann. Oncol..

[42] Ramirez A.J., Graham J., Richards M.A., Cull A., Gregory W.M., Leaning M.S., Snashall D.C., Timothy A.R. (1995). Burnout and psychiatric disorder among cancer clinicians. Br. J. Cancer.

[43] Bosshard M., Guttormsen S., Nater U.M., Schmitz F., Gomez P., Berendonk C. (2025). Improving breaking bad news communication skills through stress arousal reappraisal and worked examples. Med. Educ..

[44] Hoff L., Hermerén G. (2014). Identifying challenges to communicating with patients about their imminent death. J. Clin. Ethics.

[45] Brandes K., Linn A.J., Butow P.N., van Weert J.C. (2015). The characteristics and effectiveness of Question Prompt List interventions in oncology: A systematic review of the literature. Psychooncology.

[46] Kinnersley P., Edwards A., Hood K., Cadbury N., Ryan R., Prout H., Owen D., Macbeth F., Butow P., Butler C. (2007). Interventions before consultations for helping patients address their information needs. Cochrane Database Syst. Rev..

[47] Fujimori M., Akechi T., Morita T., Inagaki M., Akizuki N., Sakano Y., Uchitomi Y. (2007). Preferences of cancer patients regarding the disclosure of bad news. Psychooncology.

[48] Tariman J.D., Berry D.L., Cochrane B., Doorenbos A., Schepp K. (2010). Preferred and actual participation roles during health care decision making in persons with cancer: A systematic review. Ann. Oncol..

[49] Dowsett S.M., Saul J.L., Butow P.N., Dunn S.M., Boyer M.J., Findlow R., Dunsmore J. (2000). Communication styles in the cancer consultation: Preferences for a patient-centred approach. Psychooncology.

[50] Sepucha K., Ozanne E.M. (2010). How to define and measure concordance between patients’ preferences and medical treatments: A systematic review of approaches and recommendations for standardization. Patient Educ. Couns..

